# Vegetable Fungi Growing in the Human Ear—Aspergillus Nigricans or Mykomyringitis

**Published:** 1873-06

**Authors:** A. R. Kilpatrick

**Affiliations:** Navasota, Texas


### SOUTHERN MEDICAL RECORD.

VOL. III.—JUNE, 1873.—NO. 6.

### PART I.

### ORIGINAL COMMUNICATIONS.

### VEGETABLE FUNGI GROWING IN THE HUMAN EAR—ASPERGILLUS NIGRICANS, OR MYKOMYRINGITIS.

BY A. R. KILPATRICK, M.D., NAVASOTA, TEXAS.

There is no doubt that this disease is as old as any other, but from not having the aid of the microscope to detect it and show it, the profession have passed on for ages without a knowledge of it. Every revolving year, thousands of persons suffer with earache and partial deafness, and are treated empirically and in some cases relieved, while in others the disease continues indefinitely.

My attention was called to it several years ago, but the books and journals said nothing about vegetable growths in the human ear causing pain and partial deafness, but all attributed it to cold, or inflammation of the meatus or tympanum; so the usual sedatives, carminatives and hypnotics were employed. And although the aural injections which were used brought away large spongy masses, yet they were considered only cerumen which was mixed with dust, or vitiated secretions. As the removal of the abnormal masses was followed by relief each time, no more was thought about it. Having no suitable magnifying glass, or microscope, there was no examination made of them, but they were thrown away as of no consequence.

From my observation of the cases which fell under my care, and from the concurreut testimony of the authors I have read, I am satisfied that the *Aspergillus* is more frequently met with in malarial districts, or in a vitiated, damp atmosphere, than any other. I am induced, furthermore, to believe that the spores are very much the same, if not identical, with those observed by Dr. Saulsbury as being the main cause of intermittent fevers. The whole atmosphere is filled with minute vegetable particles, or spores, in countless multitudes, floating in all directions, and falling and settling in all conceivable places, which, when meeting no opposition, but happening in a suitable nidus, and being palatably nourished, spring up into the many and various parasitic forms and shapes, some of which are familiar to us.

For the successful growth of this fungus, there must be an appropriate place prepared in the auditory canal, and probably an exudative inflammation, or at least a precedent loosening and softening of the epidermis.

The symptoms attending this disease are very much the same as those of common earache caused by cold or impacted cerumen. The most prominent, however, is the roaring, or rather *singing* in the ears, *tinnitus aurium*, with a dizziness amounting almost to vertigo; pain, a sense of fullness about the ear, and a decided impairment of hearing. The pain is caused by the local inflammation, and *this* is no doubt the proper forerunner which prepares the nidus for the growth and maturation of the fungus.

Upon dilating the meatus and viewing the tympanum and walls of the canal, a close inspection will discover small flakes of a dark color and of a furry character, and the observer may suppose that it is the natural appearance of the parts. Sometimes the coating is a whitish substance which nearly fills the canal. In most of the cases the aid of a forceps is needed to remove the mass. When it is removed, the integument beneath is found reddened and sensitive.

Having said this much for the purpose of arousing Southern physicians and directing their attention to the disease, I shall proceed to detail a few well-marked cases which came under my treatment. It is to be hoped that the many improvements made of late years in the manufacture of optical instruments, their cheapness, and the facilities for learning their use, will cause, in the South, a careful study of medical mycology. When we read an essay in a Northern medical journal, we observe that the profession there are calling into use all the aids of modern improvements, and thereby advancing and increasing their scientific knowledge. We see some article detailing an account of this or that disease, and we pass it by, with the thought, "Ah, well! that is some disease peculiar to Northern climates—we have no such things here—therefore I shall not trouble myself about it."

CASE I.—Miss M. M. T., a young lady who had lived in Georgia and had suffered very much from an injury of the right ear, attended with considerable otitis, for several months. The family of which she was a member removed from Georgia in March, 1853, and while in New Orleans on their way out, she was induced to consult a Dr. Turnbull there, who was treating such diseases. The remedies he used caused much pain and suffering, without relieving the ear or improving audition. There was evidently abrasion of the meatus, and probably subacute inflammation, attended with increased aural discharge. Miss T. left the city in March, and went on up to Avoyelles parish, and, soon after reaching her destination, the intensity of the deafness was much increased, and she had the ear syringed freely every day, and dropped in glycerin. The region of country where she was is eminently paludal and malarial. She soon was attacked with dengue, which aggravated the otitis. By using the glycerin and warm injections, the mass became detached, when there was produced a feeling as though the ear was filled with a ball or some foreign substance. Upon examination by a member of the family, it was seen and removed with a pair of tweezers. It was so large as to require comminution and extracting in detached shreds and pieces. The whole mass, when placed together, was as large as the end of her thumb. After its removal she was entirely relieved, and her hearing very much improved. It presented the appearance of the *Aspergillus.*

CASE II.—This occurred in the oldest child of the foregoing, who married and during the war refugeed in Texas from the invading army under General Banks, in 1863. Her son, J. T. K., aged nine years, of general good health, while living in Tennessee Colony, in Anderson county, Texas, in the spring of 1864, was taken with severe pain in one ear, but unattended with any perceptible fever. The ear was filled with warm olive-oil and a few drops of tinct. opii, but the pain was not allayed. Warm injections of soap-suds were used in the ear and kept up for some time, when a dark mass was observed at the external meatus. Small forceps were used, and a large mass of *Aspergillus* was removed, followed by complete relief. The ear was dried and some more sweet-oil and laudanum dropped in. There was no more trouble then.

The region of country where the family lived was very subject to malarial diseases, there being as much intermittent fever and bilious fever there as in any of the swamp of Louisiana. The Trinity river runs seven miles northwest of that place, but there are only some small creeks nearer, on the east and north.

Case III.—This occurred in the same lad in 1869. The family removed from Anderson county down to Navasota, in Grimes county, after the war was over. J. T. K. was again seized with a similar pain in one ear, although he had no perceptible fever. Recollecting the history of the preceding attack in 1864, he was treated in the same way by olive-oil and tinct. opii, followed by injections of warm soap-suds in the ear, at the same time lifting the ear so as to straighten the meatus, and causing him to turn the ear down while throwing in the injections, so as to allow the free escape of what might be in the ear. The dark mass soon presented itself, and was extracted with a forceps. It was the *Aspergillus Nigricans*, and after its removal the pain entirely disappeared. Olive-oil and tinct. opii were dropped in, and a little wool inserted. There has been no recurrence of it since.

Navasota is in a decidedly malarial region, being only five miles from the Brazos river on the west, and the Navasota river two miles on the north, with lagoons and small streams nearer. Malarial diseases prevail here.

Case IV.—Miss E. P. T., sister of Case I., aged eighteen, living in Navasota; general health good. In April, 1869, she went three miles from town to spend a few days with a family living on the border of the Brazos swamp. On the third day she was attacked with deafness and singing in one ear that annoyed her that evening and night. It became painful and so annoying that her visit was shortened, and she came to town for treatment. On looking in the ear a large dark mass was easily seen. The injections were used freely and continuously, having the ear turned down over a basin and pulling up the external ear for the purpose of straightening the meatus and enlarging it. The mass became detached and presented at the orifice, and removed entire with a forceps. It was three-quarters of an inch in diameter. It was dark colored, and presented the appearance to the naked eye of a congeries of small fibres like very fine moss. The relief was complete, and there has been no return of the complaint.

If the reader has got through this far, I have no doubt he says: "I can call to mind some cases just like these, but had no idea of their true character; hereafter I shall know better what to do."

Believing, as I do, that such cases occur every year in the course of the practice of our physicians, I hope this brief report may lead them to a more careful observance and more successful treatment of them.

